# Temporal Trends in Maternal Food Intake Frequencies and Associations with Gestational Diabetes: The Cambridge Baby Growth Study

**DOI:** 10.3390/nu11112822

**Published:** 2019-11-19

**Authors:** Clive J. Petry, Ken K. Ong, Ieuan A. Hughes, Carlo L. Acerini, David B. Dunger

**Affiliations:** 1 Department of Paediatrics, University of Cambridge, Cambridge Biomedical Campus, Cambridge CB2 0QQ, UK; Ken.Ong@mrc-epid.cam.ac.uk (K.K.O.); iah1000@cam.ac.uk (I.A.H.); dbd25@cam.ac.uk (D.B.D.); 2 MRC Epidemiology Unit, University of Cambridge, Cambridge Biomedical Campus, Cambridge CB2 0QQ, UK; 3 Institute of Metabolic Science, University of Cambridge, Cambridge Biomedical Campus, Cambridge CB2 0QQ, UK

**Keywords:** pregnancy, diet, food frequency questionnaire, glucose, insulin secretion

## Abstract

Previous studies have suggested that in the first decade of this century the incidence of gestational diabetes (GDM) in pregnancy rose worldwide. In the Cambridge Baby Growth Study cohort we observed that this temporal trend was associated with an index of multiple deprivation and reductions in indices of insulin secretion. Deprivation level was not directly associated with GDM, suggesting that the temporal trend may relate more to other factors linked to it, such as dietary composition. In this study we investigated temporal trends in perceived food intake frequencies, derived from a qualitative, short questionnaire, in 865 pregnant Cambridge Baby Growth Study (CBGS) recruits. A number of food frequency ranks showed both temporal trends and associations with GDM, but of note is the frequency of egg consumption (negative temporal trend *p* = 0.03, slope = −6.2 ranks/year; negative association with GDM *p* = 3.0 × 10^−8^, slope = −0.002 increased risk/rank) as it was also positively associated with the insulin disposition index (*p* = 1.17 × 10^−3^, slope = 0.42 ranks. L/mmoL). These results are consistent with a potential protective effect of factors related to the frequency of egg consumption in pregnancy. Such factors may have contributed to the observed temporal trend in GDM risk but the overall detectable effect appears to have been small.

## 1. Introduction

Prevalence rates of gestational diabetes (GDM), traditionally defined as any form of glucose intolerance first recognised in pregnancy [1], are rising worldwide [2]. The tempo at which this is happening makes this likely to be predominantly environmentally rather than genetically mediated, although this does not exclude potential interactions between genetic variation and the environment, such as is found between dietary factors and obesity-enhancing genetic variants [3]. Environmental factors that could alter the susceptibility to GDM include changes to dietary intakes (both before and during pregnancy) and physical activity. A recent review [4] found that compliance to a Mediterranean-style diet, categorized as one with relatively higher bread, cereal, legume, vegetable, fruit, fish and olive oil intakes and lower or limited animal fat, meat and egg intakes [5], led to a reduction in GDM risk of between 15–38%. Similarly, a trial where the intervention group ate a Mediterranean diet supplemented with olive oil and pistachio nuts led to a relative risk for GDM of 73% of that of a control group fed a standard diet [6] and a similar effect in a follow-up real world (non-trial) situation [7]. Consumption of this diet was also associated with a reduced risk of other adverse effects of pregnancy [8]. In another study adherence to diet with a high alternate healthy eating index [9], a measure of diet quality that assesses conformance to federal dietary guidance, led to a reduction in GDM risk of 19–46% in a different population. In meta-analyses of studies assessing the effect of physical activity in more than 30,000 pregnancies, exercise regimes showed a reduction in GDM odds in women engaging in any type of pre-pregnancy physical activity (odds ratio 0.70 (0.57–0.85), *p* = 6 × 10^−4^, *I*^2^ = 52% (medium heterogeneity)) or in physical activity early in pregnancy (odds ratio 0.79 (0.64–0.97), *p* = 0.03, *I*^2^ = 26% (low heterogeneity)) [4]. A recent Cochrane systematic review concluded that there were reduced risks of GDM resulting from combined diet and exercise interventions during pregnancy compared with standard care [10]. Although it has been suggested that modifying dietary factors alone in pregnancy rather than prior to it may not be sufficient to alter the GDM risk very much [11], the RADIEL randomized controlled trial found that in high-risk women recruited less than 20 weeks into their pregnancy, lifestyle intervention focusing on dietary counselling, physical activity and weight gain in pregnancy was able to reduce the incidence of GDM by 39% [12]. Diet quality was improved over this time and there was lower pregnancy weight gain, perhaps not surprisingly given that there was a particular extra emphasis put on dietary factors when the ability of a study recruit to exercise was limited, such as if antenatal contractions occurred.

In the United Kingdom, as in many developed countries elsewhere [13], there has been a dietary shift since the 1970s toward diets with lower total fat contents that includes greater consumption of meats with lower fat contents such as poultry rather than pork, beef and lamb, and the drinking of semi-skimmed rather than full fat milk [14]. The reduced energy intake coming from fats induced by this dietary change has, however, not matched the decreased energy expenditure of less manual jobs and more restful leisure pursuits, contributing to a sharp rise in the prevalence of obesity [15]. This is true for pregnancy where diets in the U.K. appear to be very similar to those consumed by women who are not pregnant [16]. With temporal dietary trends and other dietary factors potentially able to alter circulating glucose concentrations, and therefore possibly GDM risk, it is conceivable that dietary factors could, at least partially, underpin changes in the incidence of GDM. Following studies in Canada [17], the United States [18,19], Israel [20] and Germany [21] all of which showed GDM becoming more prevalent in the first decade of this century, our recent analysis of women recruited to the Cambridge Baby Growth Study (CBGS) in this same decade showed a significant increase in the incidence of GDM that was associated more with a temporal reduction in indices of insulin secretion than insulin sensitivity [22]. In risk factor analysis there was a significant, albeit modestly sized, temporal trend in the index of multiple deprivation. This index was not itself directly associated with GDM, however. This led us to suggest that the temporal trend in the deprivation index could relate to GDM indirectly through changes in other factors that are themselves related to both changes in deprivation and glucose tolerance in pregnancy, such as diet [23] and exercise [24]. Therefore the primary outcome of the present study, rather than being hypothesis driven per se, was to try and find one or more food types whose frequency of consumption during pregnancy most closely reflected the temporal trend in GDM prevalence (and other indices of glucose and insulin secretion and sensitivity) that we had previously observed in the CBGS [22]. Both the year of analysis and the development of GDM were therefore our primary analyses. We found a number of statistically significant associations with these variables, generally with rather small effect sizes. These and other associations are therefore consistent with the possibility that factors related to the frequency of consumption of certain foods in pregnancy may have contributed to the observed temporal trend in GDM risk and changes in insulin secretion rates, but that the overall detectable effect was small.

## 2. Materials and Methods

### 2.1. Cambridge Baby Growth Study

The prospective and longitudinal CBGS was designed as an observational cohort initially including pregnancy, birth and infancy [25]. In its first phase of recruitment, between April 2001 and March 2009, 2229 mothers aged over 16 years of age were enrolled to it when attending early pregnancy ultrasound clinics at the Rosie Maternity Hospital, Cambridge, U.K. Of these, 571 mothers withdrew before the birth of their infant so were not considered further. Most of the clinical characteristics of the study participants were collected either during nurse-led interviews or by questionnaire with the exception of offspring birth weights, gestational ages and dates of birth, which were compiled from hospital notes. In this cohort 95.3% of the offspring were white, 1.7% were Asian, 1.3% were black (African or Caribbean) and 1.7% were other ethnicities (mainly mixed race), reflective of the population served by the Rosie Maternity Hospital [25].

### 2.2. Ethics

Ethical approval for the CBGS was granted by the Cambridge Local Research Ethics Committee, Addenbrooke’s Hospital, Cambridge, United Kingdom (00/325). All procedures followed were in accordance with the institutional guidelines. All the study participants gave written informed consent.

### 2.3. Pregnancy (Including Food Frequency) Questionnaire

A wide-ranging pregnancy questionnaire (Appendix A), that was adapted from one developed in Denmark where the self-administered form was validated through phone interviews [26], was given out to all participants at recruitment to the CBGS (around week 12 of pregnancy). Participants were requested to fill the questionnaire in as the pregnancy progressed (with assistance from research nurses if required) and the questionnaires were then collected after the birth of their baby. The questions that were asked were wide-ranging but as part of a section about lifestyle there was a short (specific) food frequency questionnaire covering most of the major food and drink types (Appendix A). For drinks, the participants were asked the number of times they drank a particular drink per day or per week (depending upon the likely consumption frequency of that drink). For food the participants were asked, ‘How often did you eat the following foods during pregnancy?’ and the response involved ticking one of the following options: never, 1–3 times per month, 1–3 times per week, 4–6 times per week or once or more per day. Participants were encouraged to tick the option that most closely resembled their food or drink intakes in pregnancy. The questionnaires were completed by 1239 of the CBGS recruits and were collected shortly after birth.

### 2.4. Oral Glucose Tests and Gestational Diabetes

At a median (inter-quartile range) of 28.4 (28.1–28.7) weeks gestation 1074 of the CBGS mothers underwent a 75 g oral glucose tolerance test (OGTT) after fasting overnight [27]. Venous blood was collected just before and 60 min after the glucose load was administered for the measurement of plasma glucose and insulin concentrations. 120 min plasma glucose concentrations were only measured from May 2007 onwards so were not used in this analysis to define GDM (only 7% of U.K. women with GDM receive a diagnosis based solely on the 120 min measurement in any case [28]). The International Association of Diabetes in Pregnancy Study Groups (IADPSG)/World Health Organization (WHO) 2013 thresholds for 0 and 60 min OGTT glucose concentrations (i.e., >5.1 and 10.0 mmol/L, respectively [29,30]) were used retrospectively to define the presence of GDM in this analysis. However when the women were recruited to the CBGS the clinical decision to treat women with GDM was broadly based on WHO 1999 guidelines [31], which considered just fasting and 2-h glucose concentrations. Based on available records and information from treating clinicians, GDM was mostly treated with diet and lifestyle modification, with or without insulin supplementation [32]. Around 19% (16 out of 83) of the women whose OGTT glucose concentrations exceeded the IADPSG/WHO (2013) thresholds for GDM [29,30] did not exceed those that were used clinically at the time and therefore did not receive dietary advice as a frontline therapy for GDM, only standard pregnancy care [32].

### 2.5. Assays

All biochemical kit-based assays were run according to the manufacturer’s instructions. Glucose concentrations were measured using a routine glucose oxidase-based method. OGTT plasma insulin concentrations were measured by ELISA (Dako UK Ltd., Ely, Cambs, UK). Intra-assay imprecision (CV) was 4.3% at 82 pmol/L, 3.0% at 402 pmol/L and 5.7% at 907 pmol/L. Equivalent inter-assay imprecision at these concentrations was 4.3, 5.1 and 5.4%, respectively.

### 2.6. Calculations

Insulin resistance and pancreatic β-cell function were estimated using the homeostasis model assessment (HOMA IR and B, respectively), calculated using the week 28 fasting circulating glucose and insulin concentrations, and the online HOMA calculator [33]. Insulin secretion (corrected for insulin sensitivity) was estimated using the insulin disposition index, calculated as the change in insulin concentrations over the first hour of the OGTT divided by the change in glucose concentrations, all divided by the reciprocal of the fasting insulin concentration. The maternal body mass index (BMI) was calculated as the pre-pregnancy weight divided by the height squared. Pregnancy weight gain was calculated as the mother’s pre-pregnancy body weight taken away from the partum body weight. The index of multiple deprivation was derived and imputed from the postcode of the participants’ home addresses as described [34].

### 2.7. Statistical Analysis and Strategy

The present analysis was restricted to those 865 pregnancies where the women underwent OGTTs (thereby excluding women with pre-existing type 1 diabetes) with 0- and 60-min plasma glucose concentrations available to us, who also completed and returned their pregnancy questionnaires. Because of being ordinal rather than continuous in nature, responses to questions about food and drink consumption frequencies were converted into ranks using standard methods before participant selection and analysis. Associations between food frequency ranks and key phenotypic variables (OGTT year, GDM, the index of multiple deprivation, HOMA IR, HOMA B, the insulin disposition index, OGTT 0 and 60 min glucose concentrations) were tested by non-parametric regression performed using the Siegel repeated medians procedure (deploying the R package ‘mblm’, version 0.12.1). Multiple testing of these non-parametric regression analyses was accounted for using the Benjamini-Hochberg procedure [35], using a false discovery rate of 0.05. Categorical analysis was performed using Fisher’s exact test. Data used in other parametric analyses were logarithmically transformed prior to analyses if the distributions of the statistical model residuals were positively skewed and transformed into reciprocals if the distributions of the residuals were negatively skewed. Further analysis was performed by standard logistic (for binary variables) or linear (for continuous variables whose model residuals using untransformed or transformed data were normally distributed) regression. Unless stated all the other statistical analyses were performed using Stata (version 13.1; Stata Corp., from Timberlake Consultants Ltd., Richmond, Surrey, UK). Statistical significance was assumed at *p* < 0.05 or lower depending on the Benjamini-Hochberg adjustment.

To be consistent with our temporal trends in GDM incidence [22] we sought statistically significant associations with both OGTT year and GDM where the slopes of the regression lines in the two models were in the same direction (i.e., either both positive or both negative). When they were, the food frequency ranks were used as confounders in logistic regression models assessing associations between OGTT year and GDM to see if they attenuated such associations. Associations with the remaining key phenotypic variables were then examined in linear regression models to assess whether they were in the same direction as the temporal trends observed in our original study [22].

To group food types that tended to be eaten with similar frequency ranks we analysed perceived food intake ranks by principal component analysis (using R version 3.6.1 (The R Foundation for Statistical Computing, Vienna, Austria), the R function “princomp” and the R packages “ggplot2” (version 3.2.0) and “factoextra” (version 1.0.5)) on a complete dataset where missing data were imputed to the median. All the R packages that we used were downloaded from http://cran.r-project.org/web/packages/mblm/index.html.

## 3. Results

### 3.1. Characteristics of Study Participants

Those women included in the present analysis tended to be representative of the CBGS cohort, albeit that on average they gave birth around 2 days later than those not included in the study (which is unlikely to have been clinically significant), were more likely to have been nulliparous and were less likely to have smoked in pregnancy (Table 1).

### 3.2. Associations with Food Intake Frequencies

#### 3.2.1. Year of OGTT (Temporal Trends)

The numbers of women who were recruited to the CBGS whose data contributed to this analysis in the nine calendar years of recruitment were: 2001→46, 2002→130, 2003→81, 2004→75, 2005→158, 2006→144, 2007→130, 2008→91 and 2009→10 (recruitment to this phase of the CBGS having closed in March 2009). Several different food and drinks showed significant temporal trends (associations between their intake frequency ranks and the year in which the pregnancy OGTT was performed (Table 2)). The largest effect size was observed in the negative association with the frequency of drinking spirits, although this is caused by only a small proportion of study participants drinking spirits with a median (and lower and upper quartile) for this population of 0 glasses of spirits drunk per week. Smaller negative temporal trends were also observed for other alcoholic drinks such as beer and wine (both with medians of zero bottles or glasses consumed per week in this population), and non-alcoholic cola (with a median (interquartile range) consumption of 0 (0, 0.5) litres per week) and fresh fruit juice (with a median intake frequency corresponding to drinking it 4–6 times per week). The only drink with a positive temporal trend was tap water. The largest significant positive effect sizes with food frequency ranks were observed with pulses (loose and canned) and tinned fruit. Comparatively much smaller, but the largest negative effect sizes were observed with food frequency ranks for eggs, canned fish and baked beans.

For clarity the temporal trends for the three dietary components with the highest absolute value for the slope (spirits, pulses and tinned fruit) are shown in bar chart form in Figure S1.

#### 3.2.2. Gestational Diabetes, Including Attenuation of the Association with Year of OGTT

A number of different food and drink intake frequency ranks showed significant (positive or negative) associations with GDM (Table 3). Not surprisingly though, none of the effect sizes of these associations were large enough to attenuate the association between GDM and year of OGTT testing. Food whose intake frequency ranks were significantly positively associated with both OGTT year and GDM (Table 2 and Table 3) were salad and fresh fruit. Equivalent significant negative associations were found with baked beans, shellfish and eggs. Drinks whose intake frequency ranks were also significantly negatively associated with both OGTT year and GDM were beer, wine and spirits. The only drink whose intake frequency was significantly positively associated with both OGTT year and GDM (albeit weakly) was tap water. The positive association between the frequency of tap water intake and GDM may seem unexpected but probably reflects the fact that the food frequency questions were designed to reflect the whole of pregnancy, not just the time preceding the development and diagnosis of GDM. Hence women diagnosed with GDM may have drunk more tap water in preference to less healthy beverages such as alcoholic drinks, in the overall context of a positive temporal trend for the frequency of tap water consumption, and negative temporal trends for beer, wine and spirits. An alternative explanation for the positive association between the frequency of tap water consumption and GDM is that polydipsia in women with poorly controlled GDM [36] could have caused them to drink tap water more often.

#### 3.2.3. Indices of Insulin Secretion & Sensitivity, OGTT Glucose Concentrations

The associations between food/drink intake frequency ranks and HOMA IR, HOMA B, the insulin disposition index, OGTT fasting and 60 min glucose concentrations (where the Benjamini-Hochberg modified *p*-value of at least one of these associations was <0.05) are shown in Table S1. Of the ranks that showed significant temporal trends and associations with GDM (in the same direction) (Table 2 and Table 3) the following also showed negative associations with indices of insulin secretion, as we observed in our original study [22]: salad, fresh fruit, tap water, wine and beer. The ranks of the frequency of the consumption of baked beans also showed a significant negative association with the insulin indisposition index, although this rules out the factors related to them having a causal role in contributing towards the temporal trend in GDM and reduced insulin secretion in the CBGS, as their intake was also negatively associated with GDM. Again, rather than reflecting factors related to them contributing towards causality, the other negative associations with the intake ranks of these specific food/drinks may be more likely to reflect dietary modifications in GDM women post-development and diagnosis. Egg intake frequency ranks were negatively associated with both year of testing and GDM, as well as positively significantly associated with the insulin disposition index. These results are consistent with either the consumption of eggs themselves, or factors related to the frequency of eggs being eaten somehow to protect against the development of GDM. Further evidence in support of this concept is gained from the fact that egg intake frequency ranks were negatively associated with both fasting and 60 min OGTT glucose concentrations.

#### 3.2.4. Index of Multiple Deprivation

Increased deprivation (a lowering of the index of multiple deprivation) was significantly associated with eating baked beans and drinking tea more frequently (the only drink whose intake frequency was significantly associated with the index of multiple deprivation), as well as eating hard cheese and white fish more frequently (Table 4). In contrast decreased deprivation was associated with eating bean curd, soya, beans/pulses, salad and organic food more frequently (amongst other foodstuffs).

### 3.3. Principal Component Analysis of Food Intake Frequency Ranks

**Figure 1:**
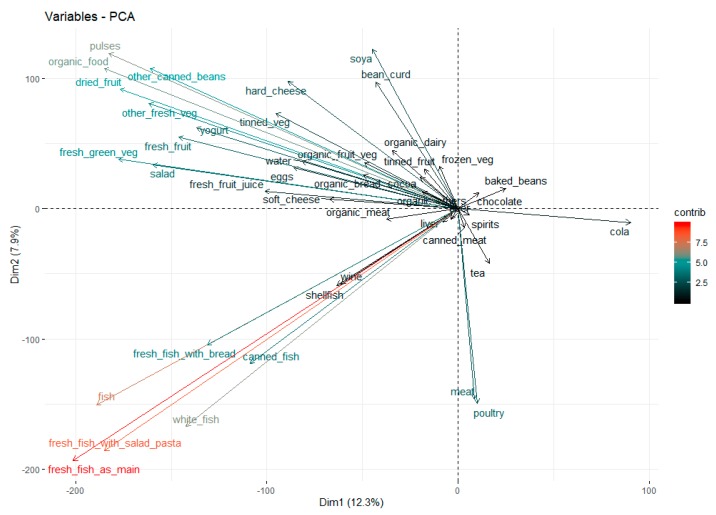
Variables plot of the first two principal components (dimensions) of maternal food intake frequency ranks in pregnancy, showing basic grouping of dietary patterns and their likely contributions to the overall variance.

Principal component analysis of maternal food intake frequency ranks in pregnancy produced 32 principal components that explained more than 1% of the variance in food intake frequency ranks. The first two principal components explained a little more than 20% of the total variance in the reported food intake frequency ranks of those food types included in the questionnaire (Figure S2). Plotting the different contributions to the first two principal components (Figure 1) (where positively correlated food intake frequencies point to the same side of the plot, and negatively correlated food intake frequencies point to the opposite side of the plot) shows several food types where the intake frequencies can be grouped (e.g., fish frequency intake in the bottom left quadrant). The frequency of egg consumption (or factors related to it), which appeared to be somewhat protective against the development of GDM in our analyses, was most closely positively related to the intake frequency of fresh fruit, salad, fresh green vegetables, tap water and yogurt. It was most negatively related to the frequency of cola intake.

## 4. Discussion

In this study we observed a number of significant associations between perceived food intake frequencies and both year of analysis (i.e., temporal trends) and GDM in the CBGS. In this cohort we had previously observed a strong positive temporal trend in the prevalence of GDM [22] and wanted to investigate the possible trends in perceived food/drink intake frequencies over the same period of time which could have contributed to this themselves or been markers of dietary factors that contributed to this. One limitation of using food intake frequencies is that associations cannot infer causality (not that changing the frequency of intake of one particular food or drink would be expected to have a big direct effect on GDM risk in any case). Indeed, of the significant food and drink intake frequencies the positive associations between frequencies of salad and tap water intakes with GDM (which also gave a positive temporal trend) are examples of food and drink types that seem particularly unlikely to have positive causal effects on GDM development. As our questionnaires were designed to be filled in across pregnancy, however, the significant positive association with salad (and tap water) intake frequency could reflect an increased frequency of consumption with GDM post-development in those women who were clinically diagnosed with the condition, in an effort for them to consume a healthier diet. Alternatively, potential confounders of this association, especially since the effect size is small, could include increased consumption of salad dressing that may be high in saturated fat and sugar content which could have slightly alter the risk of GDM [37]. Other food/drink intake frequencies showing associations in the same direction for both the year of analysis and risk of GDM include negative associations with the consumption of spirits, beer, wine, eggs, baked beans and shellfish, and a positive association with the frequency of consumption of fresh fruit. All of these associations were of modest effect sizes, and most may be more attributable to dietary modifications following a clinical diagnosis of GDM.

In the present analysis a number of perceived intake frequencies of specific foods/drinks showed temporal trends but were not associated with GDM or were associated with both but in opposite directions and could not explain the temporal trends in GDM incidence observed in the CBGS [22]. These specific foods and drink included pulses (loose or canned), fruit (dried, tinned and fresh juice), vegetables (tinned and other fresh), organic food (including fruit, vegetables, dairy, meat and others), cheese (soft and hard), fresh fish (with bread and salad pasta), yogurt, soya, bean curd, cola and chocolate. The temporal trends with the intake of these foods could relate, at least partially, to the upsurge in food prices observed in the U.K. from 2007 [38] especially in women living with a high degree of deprivation. More likely the temporal trends in the perceived intakes of pulses (loose or canned), bean curd, soya, organic food (including dairy, meat and others) and dried fruit can be explained by their associations with the index of multiple deprivation, which rose (signifying decreased deprivation) as the recruitment period for the study progressed [22]. Of the remaining food and drink types, the only one with a negative temporal trend was in the consumption of cola, which was presumably in the context of a general trend in certain populations of the U.K. for consuming healthier diets [39,40], and pregnancy diets reflecting those of non-pregnancy [16]. The observed positive temporal trends for the consumption of various forms of fruit and vegetables, and yogurt and fresh fish may also reflect the national trend for eating a healthier diet. In contrast, the positive temporal trends for chocolate and cheese intakes seem to have gone against the national trend [40,41] and may be population-specific.

With the positive temporal trend with incidence of GDM in the CBGS, a negative temporal trend was also previously observed in indices of insulin secretion [22]. There were a number of significant associations between the insulin disposition index and perceived frequencies of particular food/drink intakes in the present study. However the only food type in the pregnancy questionnaire whose frequency of intakes had statistically significant associations in directions consistent with our key phenotypes (positive with year of analysis and GDM, and negative with the insulin disposition index) were eggs, albeit in each case the associations were in the opposite direction to those associated with increased risk with time. This suggests a possible protective effect either of eggs themselves or of factors related to the frequency of egg intakes on GDM development. Consistent with the idea that is eggs themselves, at least one other study [42] has found a negative association between egg intake in pregnancy and GDM risk. However other evidence from a meta-analysis suggests that egg intakes in pregnancy may be positively related to GDM development [43] rather than protective, with the positive effect on GDM development thought to be related to one or more of the nutritive components of eggs, namely cholesterol, ω-polyunsaturated fatty acids and lutein [44]. In the present study, because of the basic (and incomplete in terms of food groups) nature of the food frequency questionnaire that was used, we analysed the food intake frequencies using simple associations with ranks, with no reference to nutritional databases to convert food intake frequencies into nutrient intakes. However we did perform principal component analysis in an attempt to group the egg intake frequencies into a basic dietary pattern. The frequencies of egg intakes appeared to be most closely positively related to intake frequencies of fresh fruit, salad, fresh green vegetables and yogurt (as may be enriched in diets such as the Mediterranean diet, which has been shown to have positive effects on reducing GDM prevalence [5,6,7]) when plotting the first two principal components of food/drink intake frequency ranks. These two components between them explained just over 20% of their variance. In the analysis by Shin et al. [37] of the National Health and Nutrition Examination Survey from around the same period of time that the CBGS participants were recruited, the biggest absolute weighting by far given for eggs was a negative one for the ‘high added sugar and organ meats; low fruits, vegetables and seafood’ dietary pattern which was associated with the highest risk for the development of GDM in pregnant women in that study. In another study from Northern Sweden, egg consumption was associated with intakes of fish, fruits, cereals and whole grain products which are noted to reduce the risk of GDM [45]. Our data from the present analysis therefore seems consistent with these two other studies [37,45], perhaps not surprisingly given the similarities in dietary habits between these countries [46]. In each case it would appear that eating more eggs might be associated with eating a ‘healthy’ diet which leads to a lower risk for the development of GDM. Using findings from the present study, plus those other cited studies [5,6,7,37,45], consumption of these ‘healthy’ rather than more standard Westernized diets would therefore appear to have beneficial effects on the development of GDM. However even with the key, highly statistically significant associations in our study, including the one with the index of multiple deprivation, and plausible explanations as to why the frequency of egg intakes in pregnancy may be related to a lower risk of developing GDM, the detectable effect size appears to be very small so will only have explained a modest proportion of the risk in the CBGS.

In the present study we analysed a short, qualitative (in terms of lack of portion size) food frequency questionnaire that was part of a much larger lifestyle questionnaire where the answers were supposed to reflect the whole of pregnancy (so in the context of GDM both pre- and post- a possible clinical diagnosis). The questions related to food and drink intake frequencies were restricted to the items listed in the questionnaire and were not designed to be comprehensive. Indeed they did not include questions related to intake frequencies of high carbohydrate foods such as potatoes and rice. Like all such questionnaires, it was therefore not reliable for assessing the total diet, energy or nutrient intakes. In addition it did not contain questions about the method of cooking of the food that was consumed. It was, however, the only form of dietary record that we had for mothers in this cohort. Another shortcoming of using the questionnaire could have been the requirement for the participants to have good recall, literacy and numerical skills, although CBGS participants tended to be relatively highly qualified [25], so this may have been less of a problem than it might otherwise have been. Participants were encouraged to fill in their questionnaires as their pregnancy progressed to further abrogate this potential shortcoming. Whilst the use of the questionnaire had a number of limitations it also had strengths. First, after the initial withdrawals from the study, 74.7% of the CBGS participants completed and returned their questionnaires so there was a relatively high response rate suggesting that the results from the present analysis were likely to be representative of those of the whole CBGS. Second, by listing specific food items (even in combination) it also made it easy for the participants to complete, which may have contributed to this high response rate. Third, by being qualitative in terms of portion sizes of specific food types eaten (but semi-quantitative in terms of food intake frequencies) the respondents did not have to estimate food intakes which could have helped limit inaccuracies (albeit by doing this they indirectly inferred that portion sizes were average and uniform). Finally, by covering the whole of pregnancy the questionnaire was not confounded by trimester.

## 5. Conclusions

Within the limitations of the short (specific) food frequency questionnaire that was analysed in this study, the food and drink intake frequency ranks with the associations that most closely resembled those linking year of analysis with GDM and related metabolic indices [22] were those linked to the frequency of egg consumption. All these relevant associations were in the opposite direction to those with the year of analysis in the CBGS, suggesting that either the frequency of egg consumption themselves or factors related to the frequency of egg intake during pregnancy (which our analysis suggests could be related to the intake of a healthier diet in pregnancy) appeared protective against GDM and reductions in insulin secretion. However the detectable effect sizes of all the individual associations were small, and certainly, these associations with GDM were not strong enough to attenuate the observed temporal trend in GDM incidence (although realistically the consumption frequencies of the single food or drink types would not be expected to do this). We conclude that in the CBGS there was a potential protective effect of dietary factors related to the frequency of egg consumption in pregnancy, a reduction in which over time could have contributed modestly to the observed temporal trend in GDM risk.

## Figures and Tables

**Table 1:** Clinical characteristics of those Cambridge Baby Growth Study participants who were included in the current analysis and those that were not.

Characteristic	Included	Not-Included	*p*-Value
Mother’s age at the birth of her baby (years)	33.4	33.7	0.3
	(33.1, 33.7)	(33.3, 34.0)	
	(*n* = 787)	(*n* = 548)	
Parity (*n*, of increasing parity and starting with 0)	415/310/103/24/4/2	302/330/112/37/4/4	2.0 × 10^−3^
GDM (*n* yes/no)	85 yes, 780 no	26 yes, 193 no	0.4
OGTT fasting glucose concentration (mmol/L)	4.3	4.4	0.1
	(4.3, 4.4)	(4.3, 4.5)	
	(*n* = 865)	(*n* = 218)	
OGTT fasting insulin concentration (pmol/L)	45	46	0.8
	(44, 47)	(44, 49)	
	(*n* = 846)	(*n* = 290)	
Pre-pregnancy BMI (kg/m^2^)	23.5	23.3	0.4
	(23.2, 23.7)	(22.9, 23.6)	
	(*n* = 776)	(*n* = 411)	
Index of multiple deprivation	8.0	8.1	0.6
	(7.7, 8.3)	(7.8, 8.5)	
	(*n* = 597)	(*n* = 401)	
Maternal highest qualification (category GCSE/A levels/degree) (*n*)	75/123/337	38/62/155	0.8
Sex of baby (*n* males/females)	443/413	412/386	1.0
Baby’s birth weight (kg) *	3.490	3.449	0.1
	(3.459, 3.552)	(3.406, 3.492)	
	(*n* = 774)	(*n* = 408)	
Gestational age at birth of baby (weeks)	39.9	39.6	5.1 × 10^−4^
	(39.8, 40.0)	(39.5, 39.7)	
	(*n* = 857)	(*n* = 800)	
Reported smoking during pregnancy (*n* yes/no)	31/825	55/744	3.0 × 10^−3^

* adjusted for maternal pre-pregnancy BMI, gestational age at birth, sex of baby and parity. Data are either the number of participants or mean (95% confidence interval).

**Table 2:** Statistically significant associations between the food/drink intake frequency ranks and year of oral glucose tolerance test (OGTT) testing in the Cambridge Baby Growth Study (CBGS) presented in descending order of the absolute value of the slope.

Food/Drink Type	Slope (Ranks/Year)	*p*-Value
Spirits	−155.06	2.12 × 10^−2^
Pulses	95.90	1.37 × 10^−24^
Tinned fruit	69.81	2.33 × 10^−5^
Other canned beans/pulses	34.29	3.27 × 10^−11^
Organic food	33.40	2.51 × 10^−12^
Beer	−32.33	2.75 × 10^−3^
Soft cheese	29.99	3.00 × 10^−11^
Tinned vegetables	29.92	1.07 × 10^−2^
Wine	−26.63	1.40 × 10^−6^
Tap water	25.25	2.63 × 10^−4^
Dried fruit	23.00	1.36 × 10^−8^
Organic fruit and vegetables	22.75	2.19 × 10^−13^
Organic dairy	19.67	2.88 × 10^−9^
Organic meat	17.98	1.07 × 10^−8^
Yogurt	17.04	5.69 × 10^−8^
Cola	−15.17	5.75 × 10^−3^
Salad	15.00	2.22 × 10^−5^
Other fresh vegetables	13.33	1.38 × 10^−3^
Bean curd	10.13	2.42 × 10^−3^
Fresh fruit	8.98	1.20 × 10^−3^
Fresh fish with bread	8.88	1.92 × 10^−3^
Hard cheese	8.41	5.54 × 10^−3^
Soya	7.05	1.68 × 10^−2^
Fresh fish with salad pasta	6.44	9.48 × 10^−3^
Eggs	−6.19	3.03 × 10^−2^
Organic others	6.13	7.16 × 10^−3^
Chocolate	5.25	2.54 × 10^−3^
Fresh fruit juice	−3.37	7.46 × 10^−3^
Canned fish	−3.28	1.64 × 10^−2^
Baked beans	−1.36	1.21 × 10^−4^
Shellfish	−1.28 × 10^−5^	2.44 × 10^−2^

*p*-values are presented unadjusted for the Benjamini-Hochberg procedure. All food types in the food frequency questionnaire that are not shown did not have a significant association with the year of OGTT testing (Benjamini-Hochberg adjusted *p* > 0.05).

**Table 3:** Statistically significant associations between the food/drink intake frequencies and GDM in the CBGS presented in descending order of the absolute value of the slope.

Food/Drink Type	Slope (Ranks/Diagnosis of GDM)	*p*-Value	Association between Year of OGTT Testing and GDM in These Women		Association between Year of OGTT Testing and GDM in These Women (Adjusted for Food/Drink Type)	
			OR	*p*-Value	OR	*p*-Value
Bean curd	−2.00 × 10^−3^	6.42 × 10^−13^	1.2	1.3 × 10^−3^	1.2	1.3 × 10^−3^
			(1.1, 1.3)		(1.1, 1.3)	
			(*n* = 890)		(*n* = 890)	
Eggs	−1.86 × 10^−3^	3.03 × 10^−8^	1.2	1.7 × 10^−3^	1.2	1.6 × 10^−3^
			(1.1, 1.3)		(1.1, 1.3)	
			(*n* = 893)		(*n* = 893)	
White fish	−1.83 × 10^−3^	3.88 × 10^−6^	1.2	1.5 × 10^−3^	1.2	1.6 × 10^−3^
			(1.1, 1.3)		(1.1, 1.3)	
			(*n* = 888)		(*n* = 888)	
Soya	−1.66 × 10^−3^	9.02 × 10^−9^	1.2	1.9 × 10^-3^	1.2	2.1 × 10^−3^
			(1.1, 1.3)		(1.1, 1.3)	
			(*n* = 890)		(*n* = 888)	
Meat	1.63 × 10^−3^	4.86 × 10^−8^	1.2	1.5 × 10^−3^	1.2	1.6 × 10^−3^
			(1.1, 1.3)		(1.1, 1.3)	
			(*n* = 893)		(*n* = 893)	
Spirits	−1.63 × 10^−3^	1.20 × 10^−17^	1.2	3.1 × 10^−3^	1.2	3.0 × 10^−3^
			(1.1, 1.3)		(1.1, 1.3)	
			(*n* = 857)		(*n* = 857)	
Beer	−1.62 × 10^−3^	1.97 × 10^−12^	1.2	3.3 × 10^−3^	1.2	4.5 × 10^−3^
			(1.1, 1.3)		(1.1, 1.3)	
			(*n* = 856)		(*n* = 856)	
Liver	−1.57 × 10^−3^	1.50 × 10^−15^	1.2	1.3 × 10^−3^	1.2	1.2 × 10^−3^
			(1.1, 1.3)		(1.1, 1.3)	
			(*n* = 889)		(*n* = 889)	
Canned meat	−1.57 × 10^−3^	4.49 × 10^−14^	1.2	1.5 × 10^−3^	1.2 (1.1, 1.3) (*n* = 892)	1.1 × 10^−3^
			(1.1, 1.3)			
			(*n* = 892)			
Poultry	1.56 × 10^−3^	7.14 × 10^−5^	1.2	1.5 × 10^−3^	1.3	1.1 × 10^−3^
			(1.1, 1.3)		(1.1, 1.3)	
			(*n* = 892)		(*n* = 892)	
Cocoa	−1.50 × 10^−3^	1.46 × 10^−6^	1.2	2.7 × 10^−3^	1.2	2.8 × 10^−3^
			(1.1, 1.3)		(1.1, 1.3)	
			(*n* = 856)		(*n* = 856)	
Fresh fish with salad pasta	−5.22 × 10^−4^	4.43 × 10^−6^	1.2	1.5 × 10^−3^	1.2	1.3 × 10^−3^
			(1.1, 1.3)		(1.1, 1.3)	
			(*n* = 877)		(*n* = 877)	
Salad	3.54 × 10^−4^	2.57 × 10^−2^	1.2	1.1 × 10^−3^	1.2	1.2 × 10^−3^
			(1.1, 1.3)		(1.1, 1.3)	
			(*n* = 881)		(*n* = 881)	
Fresh fruit	3.01 × 10^−4^	8.53 × 10^−6^	1.2	1.5 × 10^−3^	1.2	1.7 × 10^−3^
			(1.1, 1.3)		(1.1, 1.3)	
			(*n* = 895)		(*n* = 895)	
Wine	−2.99 × 10^−4^	1.74 × 10^−4^	1.2	2.0 × 10^−3^	1.2	3.9 × 10^−3^
			(1.1, 1.3)		(1.1, 1.3)	
			(*n* = 867)		(*n* = 867)	
Organic bread	−2.83 × 10^−4^	7.89 × 10^−3^	1.2	4.4 × 10^−3^	1.2	4.9 × 10^−3^
			(1.1, 1.4)		(1.1, 1.4)	
			(*n* = 603)		(*n* = 603)	
Fresh fish with bread	−2.53 × 10^−4^	2.00 × 10^−3^	1.2	1.2 × 10^−3^	1.2	1.2 × 10^−3^
			(1.1, 1.3)		(1.1, 1.3)	
			(*n* = 864)		(*n* = 864)	
Hard cheese	−2.31 × 10^−4^	1.49 × 10^−3^	1.2	1.5 × 10^−3^	1.2	1.5 × 10^−3^
			(1.1, 1.3)		(1.1, 1.3)	
			(*n* = 894)		(*n* = 894)	
Soft cheese	−2.15 × 10^−4^	5.32 × 10^−4^	1.2	3.2 × 10^−3^	1.2	3.2 × 10^−3^
			(1.1, 1.3)		(1.1, 1.3)	
			(*n* = 877)		(*n* = 877)	
Canned fish	2.13 × 10^−4^	8.82 × 10^−5^	1.2	1.5 × 10^−3^	1.2	1.6 × 10^−3^
			(1.1, 1.3)		(1.1, 1.3)	
			(*n* = 893)		(*n* = 893)	
Fresh fruit juice	1.66 × 10^−4^	2.17 × 10^−3^	1.2	1.5 × 10^−3^	1.2	1.5 × 10^−3^
			(1.1, 1.3)		(1.1, 1.3)	
			(*n* = 888)		(*n* = 888)	
Dried fruit	−1.34 × 10^−4^	5.76 × 10^−4^	1.2	1.4 × 10^−3^	1.2	7.9 × 10^−4^
			(1.1, 1.3)		(1.1, 1.3)	
			(*n* = 892)		(*n* = 892)	
Shellfish	−5.92 × 10^−5^	8.47 × 10^−3^	1.2	1.9 × 10^−3^	1.2	1.8 × 10^−3^
			(1.1, 1.3)		(1.1, 1.3)	
			(*n* = 886)		(*n* = 886)	
Tea	−5.10 × 10^−5^	1.16 × 10^−3^	1.2	2.2 × 10^−3^	1.2	2.6 × 10^−3^
			(1.1, 1.3)		(1.1, 1.3)	
			(*n* = 876)		(*n* = 876)	
Fresh fish as main course	−3.19 × 10^−5^	3.92 × 10^−4^	1.2	1.4 × 10^−3^	1.2	9.5 × 10^−3^
			(1.1, 1.3)		(1.1, 1.3)	
			(*n* = 888)		(*n* = 888)	
Baked beans	−1.55 × 10^−5^	6.05 × 10^−3^	1.2	1.5 × 10^−3^	1.2	1.1 × 10^−3^
			(1.1, 1.3)		(1.1, 1.3)	
			(*n* = 894)		(*n* = 894)	
Tap water	1.43 × 10^−5^	2.06 × 10^−3^	1.2	1.5 × 10^−3^	1.2	1.9 × 10^−3^
			(1.1, 1.3)		(1.1, 1.3)	
			(*n* = 894)		(*n* = 894)	

Data are mean (95% confidence interval where shown). OR = odds ratio. *p*-values are presented unadjusted for the Benjamini-Hochberg procedure. All food/drink types in the food frequency questionnaire that are not shown in this Table did not have a significant association with GDM (Benjamini-Hochberg adjusted *p* > 0.05).

**Table 4:** Statistically significant associations between the food/drink intake frequency ranks and the index of multiple deprivation in the CBGS presented in descending order of the absolute value of the slope.

Food/Drink Type	Slope (Ranks/Index of Multiple Deprivation Units)	*p*-Value
Bean curd	38.24	9.82 × 10^−4^
Soya	20.07	1.29 × 10^−2^
Other canned beans/pulses	18.41	4.21 × 10^−3^
Pulses	17.83	5.39 × 10^−5^
Organic food	16.36	1.20 × 10^−4^
Salad	11.58	7.05 × 10^−3^
Dried fruit	10.82	1.07 × 10^−2^
Organic dairy	10.23	2.46 × 10^−4^
Baked beans	−9.81	1.67 × 10^−3^
Tea	−9.77	1.04 × 10^−3^
Organic bread	9.47	9.65 × 10^−3^
Organic meat	9.47	1.43 × 10^−2^
Organic others	8.97	1.42 × 10^−4^
Hard cheese	−8.24	4.94 × 10^−3^
Fish	7.89	2.48 × 10^−3^
Eggs	6.70	1.56 × 10^−3^
White fish	−6.58	1.49 × 10^−2^

*p*-values are presented unadjusted for the Benjamini-Hochberg procedure. All food types in the food frequency questionnaire that are not shown did not have a significant association with the index (Benjamini-Hochberg adjusted *p* > 0.05).

## Supplementary Materials

The following are available online at https://www.mdpi.com/2072-6643/11/11/2822/s1. Table S1: Statistically significant associations between the food/drink intake frequency ranks and week 28 HOMA IR, HOMA B, the insulin disposition index and OGTT 0- and 60-min glucose concentrations in the CBGS. Figure S1: Bar charts showing the mean (S.E.M.) frequency ranks for the consumption of (a) spirits (*p*-trend = 2.1 × 10^−2^), (b) pulses (*p*-trend = 1.4 × 10^−24^) and (c) tinned fruit (*p*-trend = 2.3 × 10^−5^), all shown per year in which the 75 g OGTT was performed in the Cambridge Baby Growth Study. Figure S2: Scree plot of the first ten principal components (dimensions) of maternal food intake frequency ranks in pregnancy in the Cambridge Baby Growth Study.

## Appendix A

The prenatal questionnaire (which included the food/drink intake frequency questions) that were given out to all pregnant women recruited to the CBGS.
