# Supplementary material for: Temporal Trends in Maternal Food Intake Frequencies and Associations with Gestational Diabetes: The Cambridge Baby Growth Study
Source: Nutrients. 2019 Nov 19;11(11):2822. doi: 10.3390/nu11112822 (PMC6893826; doi:10.3390/nu11112822)

Supplementary Table and Figures to the manuscript "Temporal Trends in Maternal Perceived Food Intake Frequencies and Associations with Gestational Diabetes: the Cambridge Baby Growth Study" by Petry et al.

Table S1. Statistically significant associations between the food/drink intake frequency ranks and week 28 HOMA S, HOMA B, the insulin disposition index and OGTT 0 and 60 min. glucose concentrations in the CBGS. All food types in the food frequency questionnaire that are not shown did not have a significant association with any of these values (Benjamini-Hochberg adjusted p>0.05).

food/drink type	HOMA S		HOMA B		Insulin disposition index		OGTT fasting glucose concentration		OGTT 60 min. glucose concentration	
	**slope**	**p-value**	**slope**	**p-value**	**slope**	**p-value**	**slope**	**p-value**	**slope**	**p-value**
spirits	-3.73×10^{-2}	5.04×10^{-38}	1.12×10^{-2}	3.12×10^{-3}	7.97	3.13×10^{-12}	0	9.21×10^{-4}	3.26×10^{-4}	2.04×10^{-2}
cola	-4.19×10^{-2}	1.81×10^{-36}	2.05×10^{-2}	1.44×10^{-19}	-1.24	1.84×10^{-3}	0	3.43×10^{-3}	1.96×10^{-4}	5.08×10^{-2}
fresh fish with salad pasta	3.07×10^{-2}	3.38×10^{-33}	-1.60×10^{-2}	9.43×10^{-17}	1.86	3.90×10^{-9}	0	0.40	-2.49×10^{-4}	1.80×10^{-3}

fish	2.15x 10^{-2}	3.53x 10^{-33}	-1.49x 10^{-2}	2.80x 10^{-22}	1.54	0.74	0	0.74	-2.31x 10^{-4}	3.94x 10^{-3}
pulses	2.15x 10^{-2}	1.18x 10^{-32}	-2.07x 10^{-2}	1.32x 10^{-43}	-0.90	2.95x 10^{-2}	0	1.61x 10^{-12}	0	5.21x 10^{-2}
dried fruit	2.80x 10^{-2}	7.12x 10^{-24}	-1.71x 10^{-2}	1.26x 10^{-20}	1.30	3.88x 10^{-3}	0	3.46x 10^{-8}	-5.07x 10^{-4}	2.70x 10^{-11}
bean curd	1.48x 10^{-4}	9.75x 10^{-22}	-2.29x 10^{-2}	3.33x 10^{-34}	-0.96	6.13x 10^{-05}	2.85x 10^{-4}	3.31x 10^{-15}	-3.22x 10^{-4}	1.27x 10^{-5}
canned fish	1.91x 10^{-2}	4.67x 10^{-21}	-8.04x 10^{-3}	3.16x 10^{-6}	1.66	3.47x 10^{-11}	0	2.49x 10^{-2}	-1.87x 10^{-4}	0.14
wine	3.22x 10^{-2}	3.90x 10^{-18}	-2.12x 10^{-2}	6.63x 10^{-23}	1.52	5.69x 10^{-3}	0	5.61x 10^{-2}	-2.40x 10^{-4}	1.04x 10^{-2}
fresh fish as main	2.91x 10^{-2}	1.48x 10^{-17}	-1.44x 10^{-2}	4.41x 10^{-16}	1.24	6.26x 10^{-2}	0	0.58	-2.25x 10^{-4}	1.28x 10^{-2}

fresh green vegetables	2.30x 10^{-2}	2.15x 10^{-14}	-1.26x 10^{-2}	8.48x 10^{-9}	1.21	5.19x 10^{-3}	0	0.77	-1.18x 10^{-4}	0.13
other canned beans*	2.33x 10^{-2}	3.57x 10^{-13}	-2.16x 10^{-2}	3.47x 10^{-22}	0.20	0.15	1.83x 10^{-4}	1.32x 10^{-9}	0	5.79x 10^{-2}
yogurt	1.06x 10^{-2}	1.95x 10^{-12}	-1.33x 10^{-2}	1.33x 10^{-21}	1.62	5.84x 10^{-9}	0	1.17x 10^{-2}	0	0.29
fresh fish with bread	2.68x 10^{-2}	2.56x 10^{-11}	-1.11x 10^{-2}	1.80x 10^{-6}	0.84	0.70	0	0.18	0	0.88
meat	1.01x 10^{-2}	3.65x 10^{-11}	-1.42x 10^{-3}	0.92	1.35	1.57x 10^{-9}	0	1.09x 10^{-2}	0	4.23x 10^{-3}
fresh fruit	2.20x 10^{-2}	4.41x 10^{-11}	-8.35x 10^{-3}	1.06x 10^{-5}	1.03	4.74x 10^{-2}	0	0.83	0	0.38
other fresh vegetables**	2.15x 10^{-2}	1.03x 10^{-10}	-1.14x 10^{-2}	1.42x 10^{-8}	0.34	0.13	0	0.19	1.24x 10^{-4}	0.17

organic food	1.91x 10^{-2}	1.11x 10^{-10}	-1.56x 10^{-2}	5.48x 10^{-26}	-1.23	2.59x 10^{-3}	0	5.03x 10^{-3}	-2.55x 10^{-4}	8.82x 10^{-2}
liver	2.29x 10^{-2}	2.27x 10^{-10}	-1.55x 10^{-2}	9.74x 10^{-20}	2.43	0.72	1.57x 10^{-4}	3.31x 10^{-18}	3.14x 10^{-4}	2.38x 10^{-3}
tap water	1.97x 10^{-2}	1.64x 10^{-9}	-1.02x 10^{-2}	5.11x 10^{-6}	-7.74x 10^{-2}	0.71	0	4.85x 10^{-3}	1.64x 10^{-3}	2.33x 10^{-2}
tinned vegetables	1.87x 10^{-2}	1.15x 10^{-7}	-4.96x 10^{-4}	3.16x 10^{-3}	0.11	0.99	0	4.40x 10^{-2}	0	0.96
soft cheese	1.74x 10^{-2}	6.07x 10^{-7}	-1.18x 10^{-2}	1.64x 10^{-9}	-7.04x 10^{-2}	0.15	0	0.80	0	0.32
hard cheese	9.38x 10^{-3}	1.08x 10^{-6}	-7.53x 10^{-3}	1.16x 10^{-7}	1.24	0.30	-1.98x 10^{-4}	2.53x 10^{-7}	0	6.63x 10^{-2}
baked beans	-6.47x 10-3	4.71x 10^{-6}	1.68x 10^{-3}	0.23	-0.96	5.90x 10^{-5}	0	0.96	3.81x 10^{-4}	1.10x 10^{-3}

eggs	4.34x 10^{-3}	6.89x 10^{-6}	-1.87x 10^{-4}	4.13x 10^{-2}	0.42	1.17x 10^{-3}	-9.80x 10^{-5}	5.14x 10^{-6}	-3.74x 10^{-4}	1.77x 10^{-8}
chocolate	-1.64x 10^{-4}	1.51x 10^{-5}	0.02	8.57x 10^{-6}	-3.29	2.38x 10^{-2}	0	0.67	0	0.03
white fish	2.02x 10^{-2}	6.56x 10^{-5}	-7.74x 10^{-3}	1.78x 10^{-6}	1.29	8.42x 10^{-2}	0	5.31x 10^{-2}	0	0.30
frozen vegetables	-9.48x 10^{-3}	1.46x 10^{-4}	8.91x 10^{-3}	6.68x 10^{-6}	0.34	0.59	0	2.91x 10^{-2}	2.29x 10^{-4}	1.29x 10^{-2}
tinned fruit	-9.46x 10^{-3}	3.56x 10^{-4}	1.29x 10^{-2}	2.17x 10^{-11}	-0.55	0.12	-1.16x 10^{-4}	2.05x 10^{-6}	1.79x 10^{-4}	0.32
salad	1.69x 10^{-2}	1.90x 10^{-3}	-7.23x 10^{-3}	1.62x 10^{-5}	-0.45	1.29x 10^{-3}	0	0.86	1.36x 10^{-4}	4.88x 10^{-2}
organic others***	-2.41x 10^{-2}	6.27x 10^{-3}	1.86x 10^{-2}	1.29x 10^{-2}	-1.83	0.32	0	0.19	-8.69x 10^{-4}	1.39x 10^{-2}

organic fruit/ vegetables	-1.57×10^{-2}	1.20×10^{-2}	7.97×10^{-3}	9.39×10^{-2}	-1.12×10^{-2}	0.74	0	0.30	-4.60×10^{-4}	3.16×10^{-3}
cocoa	-3.04×10^{-3}	1.31×10^{-2}	5.74×10^{-3}	0.19	0.75	0.24	0	7.81×10^{-3}	1.59×10^{-4}	0.16
beer	9.64×10^{-3}	3.45×10^{-2}	-1.07×10^{-2}	1.54×10^{-8}	0.83	0.18	0	0.93	-3.23×10^{-4}	3.87×10^{-6}
tea	-7.51×10^{-4}	0.67	-2.19×10^{-3}	0.66	2.04	2.28×10^{-7}	0	0.91	-4.62×10^{-4}	6.05×10^{-8}
soya	3.11×10^{-3}	0.24	-2.11×10^{-4}	0.07	-1.43	2.63×10^{-4}	0	4.81×10^{-2}	-1.41×10^{-4}	0.30
poultry	0	0.11	2.17×10^{-3}	2.06×10^{-5}	0.13	6.82×10^{-4}	0	6.45×10^{-3}	2.07×10^{-4}	1.09×10^{-10}
organic dairy	1.47×10^{-3}	0.71	-3.52×10^{-3}	4.55×10^{-2}	-2.85	7.45×10^{-3}	0	3.79×10^{-3}	-3.31×10^{-4}	0.04

shellfish	3.07×10^{-3}	0.79	-4.04×10^{-3}	4.74×10^{-2}	-0.24	2.84×10^{-2}	1.62×10^{-4}	7.94×10^{-10}	3.23×10^{-4}	4.07×10^{-4}
organic meat	9.82×10^{-3}	7.05×10^{-2}	0	0.85	-0.61	0.63	0	9.98×10^{-3}	-7.50×10^{-4}	1.63×10^{-5}
organic bread	-5.68×10^{-4}	0.88	7.98×10^{-3}	1.41×10^{-2}	0.72	0.14	0	0.23	-8.52×10^{-4}	1.41×10^{-5}

*Other canned beans or pulses – not baked beans.

**Other fresh vegetables – not green vegetables, e.g. turnip, swede, carrots, onions

***Other organic – origanic or home grown food

Figure S1. Bar charts showing the mean (S.E.M.) frequency ranks for the consumption of (a) spirits (p-trend=2.1×10^{-2}), (b) pulses (p-trend=1.4×10^{-24}) and (c) tinned fruit (p-trend=2.3×10^{-5}), all shown per year in which the 75 g OGTT was performed in the Cambridge Baby Growth Study.

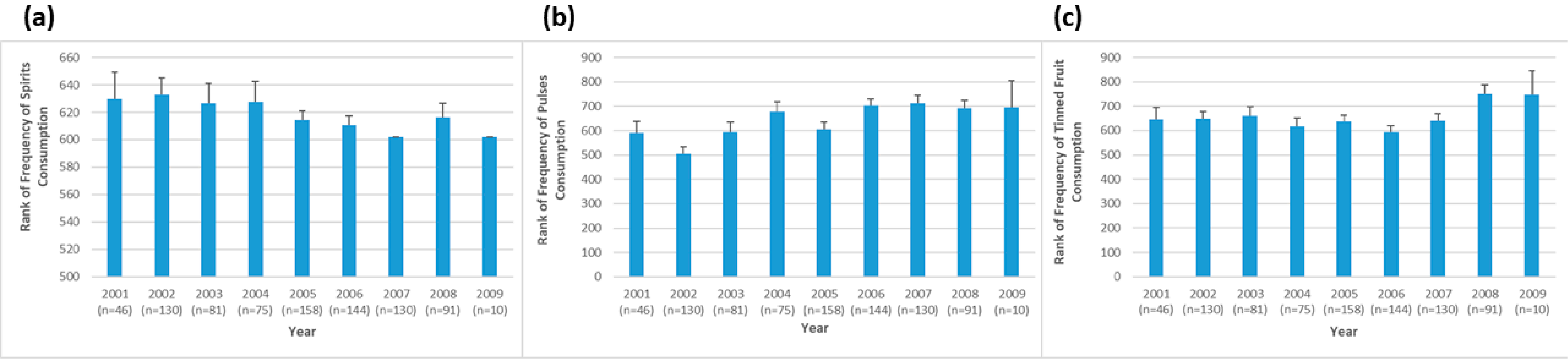

Figure S2. Scree plot of the first ten principal components (dimensions) of maternal food intake frequency ranks in pregnancy in the Cambridge Baby Growth Study.

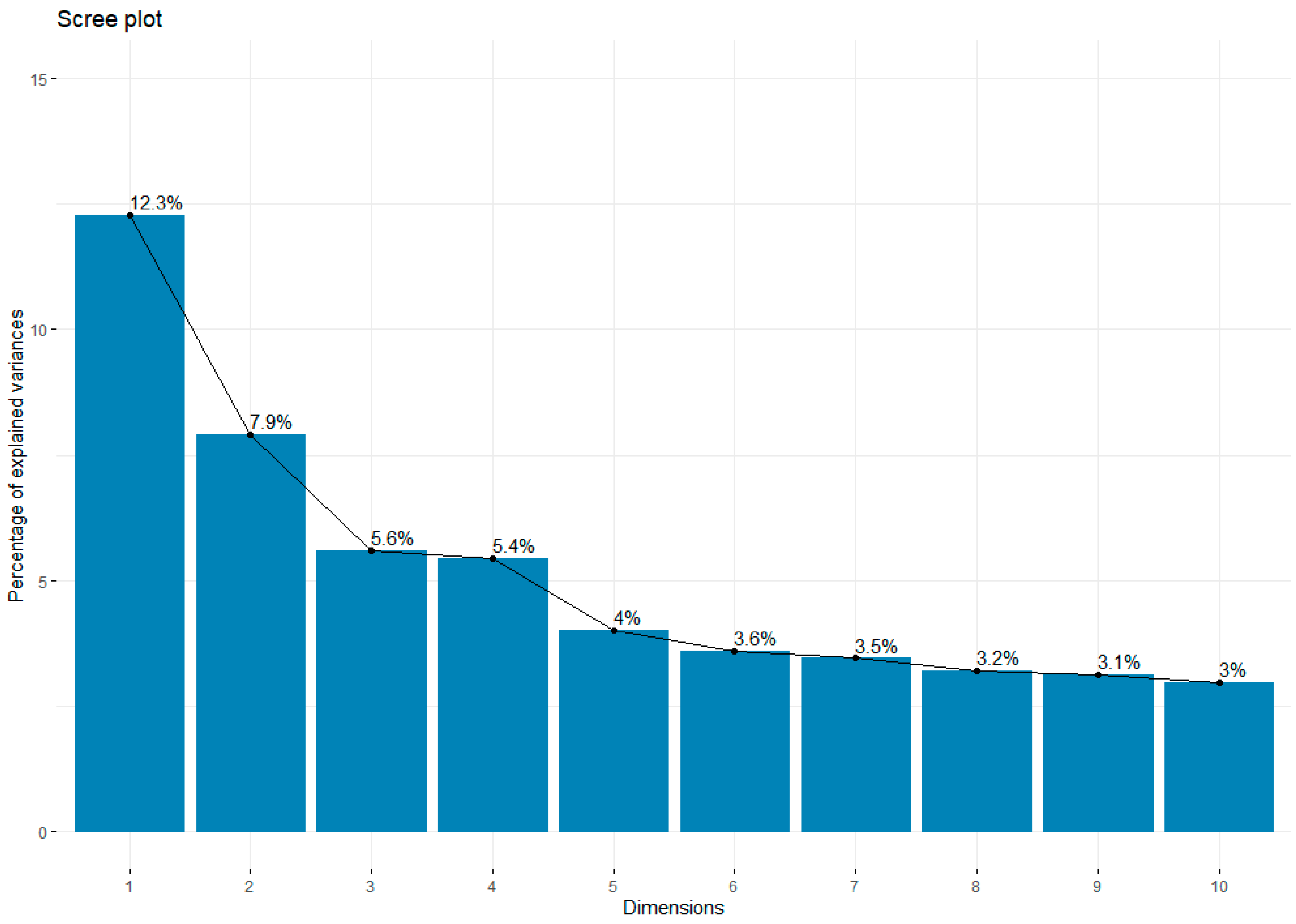

Supplement: Supplementary file 1 [file nutrients-11-02822-s001.pdf]
